# Establishment of an Automatic Real-Time Monitoring System for Irrigation Water Quality Management

**DOI:** 10.3390/ijerph17030737

**Published:** 2020-01-23

**Authors:** Wei-Jhan Syu, Tsun-Kuo Chang, Shu-Yuan Pan

**Affiliations:** Department of Bioenvironmental Systems Engineering, National Taiwan University, Taipei City 10617, Taiwan; water701009@yahoo.com.tw (W.-J.S.); tknchang@ntu.edu.tw (T.-K.C.)

**Keywords:** heavy metals, stripping voltammetry, data imputation, cloud technology, illegal discharge, improvement strategies

## Abstract

In order to provide the real-time monitoring for identifying the sources of pollution and improving the irrigation water quality management, the integration of continuous automatic sampling techniques and cloud technologies is essential. In this study, we have established an automatic real-time monitoring system for improving the irrigation water quality management, especially for heavy metals such as Cd, Pb, Cu, Ni, Zn, and Cr. As a part of this work, we have first provided several examples on the basic water quality parameters (e.g., pH and electrical conductance) to demonstrate the capacity of data correction by the smart monitoring system, and then evaluated the trend and variance of water quality parameters for different types of monitoring stations. By doing so, the threshold (to initiate early warming) of different water quality parameters could be dynamically determined by the system, and the authorities could be immediately notified for follow-up actions. We have also provided and discussed the representative results from the real-time automatic monitoring system of heavy metals from different monitoring stations. Finally, we have illustrated the implications of the developed smart monitoring system for ensuring the safety of irrigation water in the near future, including integration with automatic sampling for establishing information exchange platform, estimating fluxes of heavy metals to paddy fields, and combining with green technologies for nonpoint source pollution control.

## 1. Introduction

The best agricultural practices would be based on the transmissions and fates of pollutants in the agricultural environments, including land use planning, irrigation water quality monitoring, sediment management, soil quality maintenance, crop safety testing, and even the disclosure of production and sales information [1]. In response to the new demands and challenges, the management technical support and the coaching mechanism have been effectively integrated with the irrigation water quality management system in Taiwan. This system has thus achieved significant improvements in the performance of water conservation and water quality management businesses [2]. In addition to the existing management measures, more supporting efforts would be extended to achieve the goal of counselling and strengthening irrigation water quality monitoring and management operations, while harmonizing irrigation water quality protection policies to ensure the security and safety of food supply [3].

According to the available statistic data in 2018, the total agriculture area in Taiwan is approximately 791,000 hectares [4], accounting about 22% of the total territory of Taiwan. The rapid expansion of industries and the increase of urban settlements together with the issue of lacking integrated planning in regional irrigation and drainage have resulted into the interruption of agricultural irrigation channels by urban drainage (sewer) systems. This would cause severe water quality issues, such as heavy metals pollution in irrigation ditches. Deterioration of irrigation water quality because of the heavy metals have led to the detriment of agriculture land and the downstream safety of food supply chains [5]. Sometimes, the introduction of heavy metals into irrigation water ditches was attributed to illegal discharges of industrial wastewater at a random period of time (often at nighttime, holidays, or during heavy rains), which was difficult to inspect and manage. In Taiwan, the types of industries in close proximity to watershed (e.g., irrigation water ditch) determines the types of heavy metals deteriorating the quality of irrigation water. According to the report of Council of Agriculture, Taiwan [6], heavy metals including cadmium (Cd), lead (Pb), copper (Cu), nickel (Ni), zinc (Zn), and chromium (Cr) are commonly detected in waterbody of an irrigation ditch in Taiwan. Measuring the concentrations of trace heavy metals in bioenvironmental interfaces such as water and soil, and even in food science has always been a vital part of modern environmental monitoring. It is expected that commercial products for water quality monitoring should be cost-effective, accurate, simple to use, and easy to integrate into existing systems.

In order to overcome this critical issue, several innovative inspection technologies, such as time-lapse capsules [7,8], have been developed and deployed in the field tests for identifying the patterns of the heavy metals from the illegal discharge. Apart from the innovative inspection, the integration of continuous automatic sampling techniques and cloud technologies was imperative to provide the real-time monitoring for identifying the sources of pollution and improving the irrigation water quality management. An instant response and action to on-going illegal discharge could be taken to avoid the subsequent continuous pollution in agricultural water resources. Existing manual sampling exhibited difficulties in real-time water quality monitoring, in terms of frequency and intensity of sampling [9]. The development and deployment of continuous automatic sampling and real-time water quality monitoring system were important to overcome the aforementioned issue. It could not only generate the information on the changes in real-time water quality under different time periods and weather conditions, but also provided the opportunities for the inspection of illegal discharge of industrial wastewater in upstream. Therefore, instant responses to the illegal discharge, such as early warnings, immediate actions and amelioration, could be taken within a short time.

To the best of our knowledge, little-to-no research has been reported on the real-time monitoring of terrestrial water quality in Taiwan to realize the sustainable irrigation water management. In this study, we have developed the automatic real-time monitoring system for improving the irrigation water quality management, especially inspecting illegal discharge of industrial wastewater. For this purpose, we have first provided several examples regarding the basic water quality parameters, including pH and electrical conductance (EC), to demonstrate the capacity of data correction for the smart monitoring system. Then, we have evaluated the trend and variance of water quality parameters (e.g., EC) for different types of monitoring stations. We have also provided the representative results from the real-time automatic monitoring system of heavy metals, including Cd, Pb, Cu, Ni, Zn, and Cr, from different monitoring stations at Taoyuan, Taichung, Changhua, and Kaohsiung. Finally, we have demonstrated the applications of the developed smart monitoring system for ensuring the safety of irrigation water in the near future. This research should be considered as the pioneering study reporting the results of on-site water quality monitoring in Taiwan.

## 2. Materials and Methods

### 2.1. Establishment of Automatic Real-Time Monitoring System

**Figure 1:**
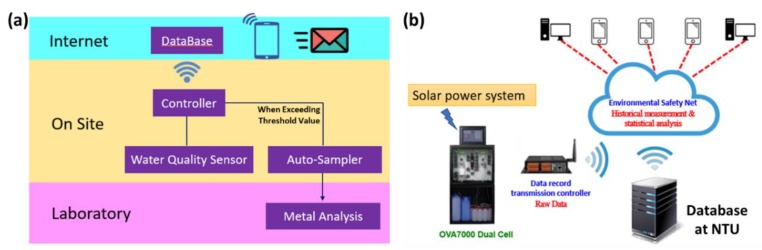
(**a**) Framework of general operations for automatic real-time monitoring systems based on cloud technology; (**b**) real-time automatic monitoring of heavy metals, powered by the solar power system.

In this study, in order to detect the basic water quality parameters and the concentration of various heavy metals, we have developed and established the automatic real-time monitoring system based on the electrochemical-approach analysis and cloud technology, as shown in Figure 1a. The major components of the real-time water quality monitoring stations consist of (i) cloud technology and database, (ii) auto-sampling and on-site sensors, and (iii) laboratory analyses of representative water samples. For instance, the transmission of real-time heavy metals monitoring data is illustrated in Figure 1b. The real-time automatic monitoring of heavy metals is powered by the solar power system, and the database of heavy metals monitoring is installed at the Irrigation Water Quality Control Center, National Taiwan University. The statistical analysis of historical measurement for irrigation water quality, including heavy metals, is open access to publics, which can be easily operated through 3C devices.

**Figure 2:**
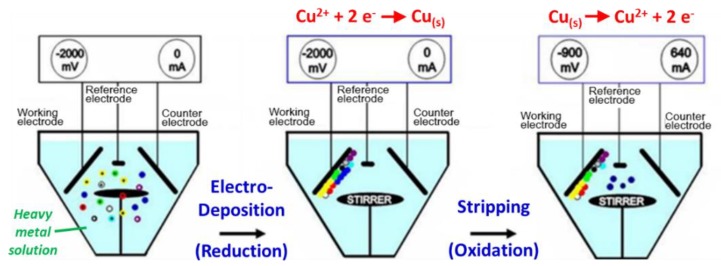
Three-stage reactions of dissolution voltammetry for monitoring heavy metals in water, exemplified by copper ions in water. Adapted from the report [6].

For heavy metals, the available on-site automatic analyses were based on either the spectroscopic or electrochemical (stripping voltammetry) methods. The spectroscopy-based method could be easily influenced by various factors, such as chromaticity and turbidity [10], and thus could be difficult in analyzing different heavy metals simultaneously in the field. In contrast, the stripping voltammetry method is easily adaptable to the on-site, portable analysis of heavy metals with sub-ppb limits of detection [11]. Therefore, in this study, we have used the stripping voltammetry (OVA7000 Dual Cell, Modern Water, the UK) to conduct the real-time automatic monitoring of heavy metals. The stripping voltammetry method consists two major steps for determining the concentration of heavy metals in water sample, as shown in Figure 2: (i) electrodeposition, or so-called pre-concentration, and (ii) stripping. In the electrodeposition step, the cathodic reduction of dissolved metal ions (M*^n+^*_(aq)_) in the water sample occurs to form metallic deposits (M_(s)_) on the surface of the electrode, as shown in Equation (1). Then, the anodic oxidation (so-called stripping) of the metallic deposits back to metal ions occurs, as shown in Equation (2), to determine the concentration of metal ions using the current-potential curve.

M*^n+^*_(aq)_ + *n* e^‒^ → M _(s)_ (1)

M _(s)_ → M*^n+^*_(aq)_ + *n* e^‒^ (2)

Since the different heavy metals had different elution potentials, the concentrations of heavy metal ions in the solution could be determined by analyzing the eluted current peak obtained from the voltammetric spectrum (Figure S1). The height of each elution current peak would be proportional to the concentration of the metal ions in the solution [12]. Then, the concentrations of heavy metals determined from the voltammetric spectrum will be uploaded to the Internet database (see Figure 1b)**.**

In this study, we compiled the commercially available sensors for measuring water quality to develop an automatic real-time monitoring system, and then installed the developed system in the field for continuous monitoring. Table 1 presents the specification of the commercial sensors, including pH, temperature (Temp.), EC, and heavy metals, used in the automatic real-time monitoring system. The sensors for measuring water quality could be applied for water samples from rivers, lakes, groundwater, and even industrial wastewater. The ranges of measurement for pH, Temp., and EC are 0‒14, −5‒50 °C, and 0‒100 mS/cm, respectively. The sensors for pH, Temp., and EC were directly placed in waterbody, and the frequency of data generated is 1 min. The detection of heavy metals is based on the stripping voltammetry method, where the working and counter electrodes were glass/carbon and platinum, respectively, with the reference electrode of Ag/AgCl in KCl solution. About 10 mL of water was automatically sampled every 30 min for the on-site analysis of heavy metals by the stripping voltammetry. The sensors could detect a range of heavy metals, such as Cd, Pb, Cu, Ni, Zn, and Cr. The limits of detection of heavy metals could reach several μg/L: Cd (0.5 μg/L), Pb (1.0 μg/L), Cu (1.0 μg/L), Ni (2.0 μg/L), Zn (10.0 μg/L), and Cr (10.0 μg/L). The time for analyzing each heavy metal using the stripping voltammetry method is illustrated as follows: Ni (10 min), Zn (10 min), Cr (15 min), Cd (20 min), Pb (20 min), and Cu (20 min). If we take the heavy metal as an example, the numbers of data for a single heavy metal gathered from a station in one year will be more than 17,000. Because of these large number of data, in this study, we provided several representative monitoring results that could demonstrate the capacity of identifying unusual events by the automatic real-time monitoring system.

### 2.2. Locations of Basic Water Quality Stations

**Figure 3:**
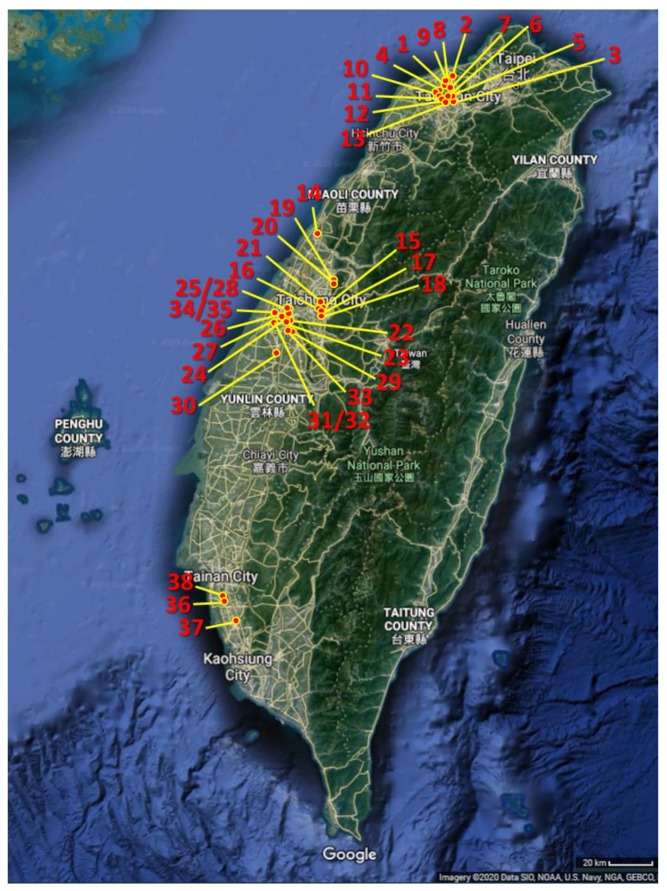
Representative automatic monitoring networks for irrigation water quality monitoring at Taoyuan, Taichung, Changhua, and Kaohsiung. The satellite map is from Google Maps (Map data©2020 Google; https://www.google.com/maps/place/Taiwan). The maps were edited with PowerPoint (version 1808).

In this study, we have developed and designed the automatic monitoring networks for irrigation water quality monitoring. Table 2 presents the representative stations of automatic monitoring systems equipped with these basic water quality sensors, as well as associated with the heavy metal monitoring. Automatic monitoring systems (stations) with water quality parameters, such as pH, EC, water level, and temperature, were installed in the field. By 2018, we have successfully installed a total of 61 automatic monitoring stations at Taoyuan, Taichung, Changhua, and Kaohsiung, as shown in Figure 3. It is noted that these monitoring stations cover around 8700 hectares of agriculture irrigation areas [6]. Figure S2 (see Supplementary Information) shows the representative photos of automatic monitoring stations at Taoyuan, Changhua, and Kaohsiung. In Taiwan, because the industries are in close proximity to irrigation water ditch, heavy metals such as Cd, Pb, Cu, Ni, Zn and Cr pose a potential threat to the quality of irrigation water. Therefore, pH, EC, water temperature *(T_m_*), water level, and heavy metals (e.g., Cd, Pb, Cu, Ni, Zn and Cr) are the monitoring parameters of water quality. We designed different threshold values of pH and EC for each station, where automatic sampling function would initiate once the pH or EC exceeds the threshold values. The sampled water would be carefully stored, and then analyzed in the laboratory to provide the evidence of unusual events, such as illegal discharge. In Taiwan, the Irrigation Water Quality Standard for pH and EC is 6‒9 and <750 µS/cm, respectively [13]. Therefore, the threshold of pH for all monitoring stations is designated to be within the range of 6 and 9. However, for the threshold of EC, since the background concentration of EC in natural waterbody varies from place to place, we develop two different approaches for designating the threshold based on the historical monitoring data of EC. If the EC in natural waterbody is mostly lower than 750 µS/cm (i.e., the Irrigation Water Quality Standard), the threshold of EC for this station is set to be 750 µS/cm. If not, we analyzed the historical continuous monitoring data (for two weeks) and then utilized the secular trend method to determine the threshold of EC. In this study, the criteria of the secular trend method are to designate a threshold value, where the unusual events in the past week occur for less than two times.

### 2.3. Data Acquisition, Collection, and Correction

Inaccurate data of water quality parameters might be generated because of the environmental disturbances, instrumental errors, and bad electrode conditions. Therefore, the regular calibration and maintenance of the sensors and equipment must be performed. For the maintenance, we developed a standard procedure of cleaning by either electrochemical or chemical methods, depending upon the conditions of field events and incidences. We schedule an automatic procedure using the electrochemical method (the reverse-current cleaning) every 45 days to periodically remove the biofouling on the electrode. In the case of chemical cleaning (manual), we used 0.1 M NaOH (or 0.1 M HCl for worse cases) to remove the contaminants on the surface of the electrode (sensor). We optionally used acetone or alcohol for cleaning if being contaminated by oil. On the other hand, the missing data and/or spectrum images might be amended by various types of data impute techniques. In this study, we have followed the guidelines and standards suggested by the U.S. Geological Survey (USGS) [14] for automatic water quality monitoring. For instance, the errors of the automatic monitoring data (*E*) were typically attributed by two factors: the fouling error (*E_f_*) and the offset error (*E_d_*), as described by Equation (3):

(3) $$E=\left|E_{f}\right|+\left|E_{D}\right|$$

In this study, we have applied two different approaches for the corrections of the fouling error. It was stated when the water quality was stable during the maintenance period, the correction coefficient of the fouling error (*C_f_*) can be determined by Equation (4):

(4) $$C_{f}=M_{a}-M_{b}$$

where *M_a_* is the post-cleaning monitoring value, and *M_b_* is the pre-cleaning monitoring reading. However, the conditions of real environments were stable because of various factors, such as thermal effects and biological reactions. It was discussed when the water quality was unstable during the maintenance period, the correction coefficient of the fouling error (*C_f_*) can be determined by Equation (5):

(5) $$C_{f}=\left(M_{a}-M_{b}\right)-\left(F_{e}-F_{s}\right)$$

where *F_e_* is the field meter reading at the end of the equipment maintenance, and *F_s_* is the field meter reading at the beginning of the equipment maintenance.

## 3. Results and Discussion

### 3.1. Data Correction for Basic Water Quality Parameters: pH and EC

**Figure 4:**
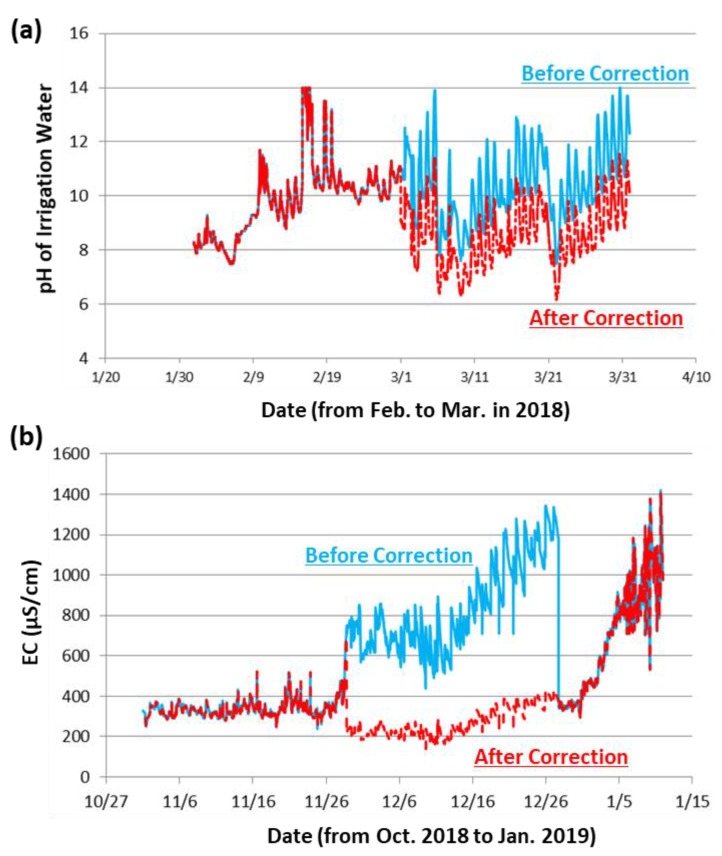
(**a**) Trend of pH value before (solid, light blue color) and after (dash, light red color) correction: in the case of the drainage station of Sanjia Village, Changhua; (**b**) trend of electrical conductivity (EC) value before (solid, light blue color) and after (dash, light red color) correction: in the case of Taichung Babao Irrigation Ditch.

For measurement conducted in the field, fouling (due to adsorption of bacteria and contaminants, such as organic matter and oil) and/or scaling (such as calcium carbonate) could be formed on the surface of electrode. The condition of the electrode is critical to ensure the data quality of each measurement. Here, we have provided several examples regarding the basic water quality parameters, including pH and EC, to demonstrate the capacity of data correction for the smart monitoring system. For instance, we have corrected the measured pH values from February 2018 to March 2018 at the Sanjiacun Drainage Station, Changhua using Equation (6). Figure 4a shows the monitoring data of the pH values before and after the correction.

(6) $$\%C_{f}=\frac{\;\left(\;8.5\;-\;10.3\;\right)\;-\;0}{10.3}\times 100=-17.4$$

We have also corrected the measured EC values from October 2018 to January 2019 at the Taichung Babaozhen Station. The original EC value before correction was 1184 μS/cm, while the value after cleaning was found to be 381 μS/cm. Figure 4b shows the monitoring data of the EC values before and after the correction. Since the water quality at this station was relatively stable, the data correction was performed by the gravimetric error percentage correction method (Equation (7)) to refine the measured EC value.

(7) $$\%C_{f}=\frac{\;\left(\;381-\;1184\;\right)\;-\;0}{1184}\times 100=-67.8$$

### 3.2. Trend and Variance of Water Quality Parameters for Different Types of Monitoring Stations: Exemplified by Electrical Conductance

**Figure 5:**
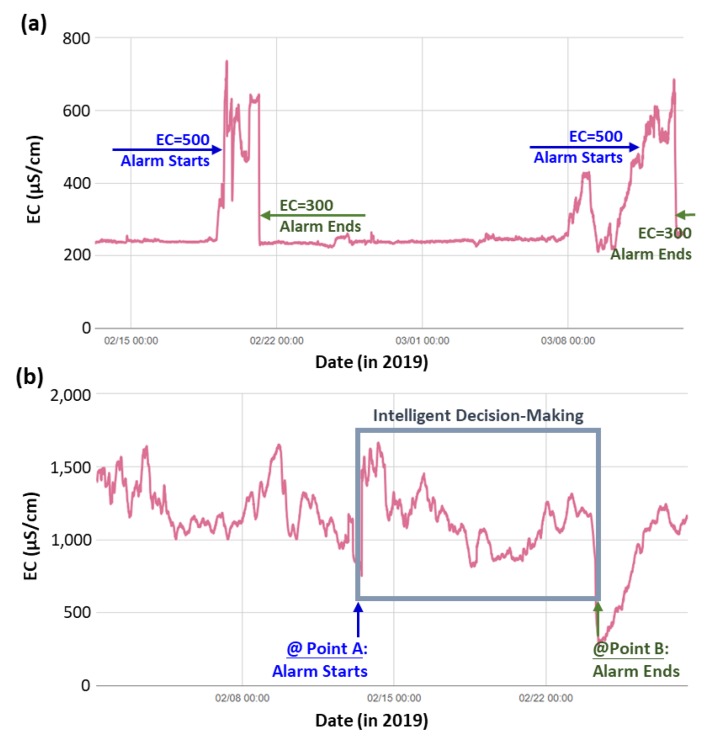
Threshold management of the measured EC values (**a**) for source-water type monitoring station at Taoyuan, and (**b**) for ditch-water type monitoring station at No. 25-3 Water Guide Station in Taoyuan Sanquaichuo Branch.

Here, we have evaluated the trend and variance of EC values for different types of monitoring stations. The monitoring stations could be generally categorized into two types, namely the source-water type station and the ditch-water type station. The source-water type stations usually exhibit the features of a large water quantity with stable water quality, while the ditch-water type stations have relatively smaller water flow rates with larger variances in water quality parameters. Since the variations of EC for the source-water type station was relatively small (stable water quality), the threshold of its EC management was mainly based on the observed trends. In this study, we have selected a certain period (usually from two weeks to one month) for the trend evaluation. During this certain period, any unusual peak of EC value (associated significant changes in the peak areas) would be automatically identified by the systems. Figure 5a shows a case study of managing the EC threshold for a source-water type monitoring station. In this example, we set the value of EC threshold as two times higher than the usual observation of EC value (i.e., ~250 µS/cm). Once the EC value from the on-line sensors exceeded the threshold (500 µS/cm), the monitoring system would issue an alarm until the on-line measurement was lower than 300 µS/cm.

In the case of a ditch-water type station, the EC variations would be much larger because of its complex water quality, as shown in Figure 5b. Therefore, to set up an appropriate threshold for EC values, we have used the moving average rate of change instead of a single limit. A high moving average rate of change usually represented an unusual event, such as illegal discharge of wastewater. We set the threshold of EC values as 30‒50% of the 1-h moving average rate of change. Once the measured EC value was found to be higher than this threshold, an alarm would be issued until the measured EC value was lower than the 1-h moving average rate of change. It was noted when the 1-h moving average rate of change was dynamic, and thus the periodic change would be- automatically calculated by the system based on the past data. In this example (see Figure 5b), at the Point A, the EC rapidly increased to 1435 µS/cm at the time of 12:20. The 1-h moving average of EC was about 800 µS/cm, and the rate of change of 1-h moving average of EC was 44.3%, exceeding the default threshold value (i.e., 30%). At the Point B, the EC decreased to 591 µS/cm, and the rate of change of 1-h moving average of EC became negative. One thing should be noticed that, except for the illegal discharge, the EC value detected by the sensor may be changed by various factors, such as erosion of terrestrial soil and fouling on the electrode surface. The purpose of the alarm was to notify the authorities about the unusual events (e.g., illegal discharge), where subsequent decisions and actions should be immediately taken.

In the past, most investigation and evaluation work of water pollution rely on regular water quality inspections to identify the unusual events, such as illegal discharge of heavy metals, by the changes in concentration. It is noted that the illegal discharge of heavy metals will result in the changes in basic water quality parameters, including pH and EC. The hydrogeological characteristics of the water channels and/or ditches would also affect the transmission behaviors of heavy metals in waterbody. Therefore, automatic real-time monitoring for basic water quality parameters (e.g., pH and EC) is effective to immediately notify the unusual events in a certain waterbody, instead of regular sampling and analysis of heavy metals. According to our experience, natural disturbance, such as soil erosion and seasonal temperature difference, would also result in minor fluctuations of EC data (within ±30% difference). In the case of illegal discharge, the EC data may significantly increase from at least 30% up to even 3 folds.

### 3.3. Real-time Automatic Monitoring for Heavy Metals

**Figure 6:**
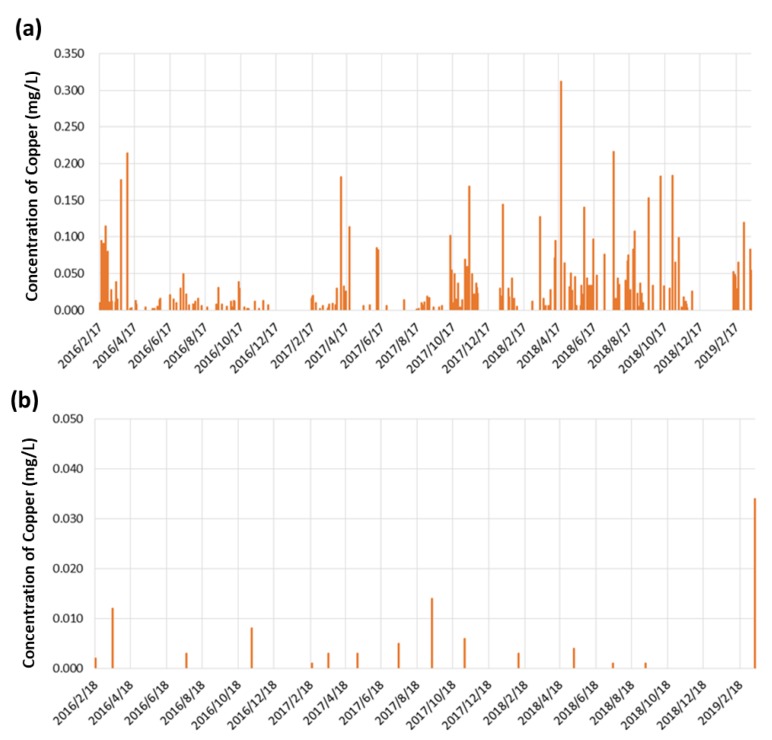
Comparison of (**a**) automatic sampling at Tieshan branch line, and (**b**) regularly manual sampling at Tieshan branch line for monitoring Cu concentration of irrigation water.

We have developed a portable automatic sampling equipment powered by a solar panel, as shown in Figure S3 (see Supplementary Information), to collect the water samples every three hours. We have also designed a novel approach to road-ground installation instead of the conventional inner-side (waterbody) installation. Figure 6 shows the comparison of the monitoring results from the developed automatic sampling equipment and that from the regular manual sampling approach. This information was collected at the East/West Sanshan-Tieshan-branch (ditch-water type) station during February 2016 to March 2019. The results indicated that the automatic sampling approach (Figure 6a) was much informative when compared to the manual sampling (Figure 6b) The automatic sampling approach was able to reflect the real situation of heavy metals in waterbody. According to the results of automatic sampling, we have also found that the concentrations of copper varied significantly at different times. The concentrations of copper in waterbody ranged from ~0.01 mg/L to even more than 0.30 mg/L. It was noted that the manual sampling with subsequent analyses in a laboratory (e.g., using inductively coupled plasma with optical emission spectrometry) could provide capability to detect heavy metals at ppt level [11]. However, it was difficult to gather the water samples at the moment in which the unusual events, such as illegal discharge, were occurring. Despite their higher accuracy and precision in data quality compared to automatic sampling, the manually collected samples mostly reflect the natural concentration of the heavy metals in the water body.

**Figure 7:**
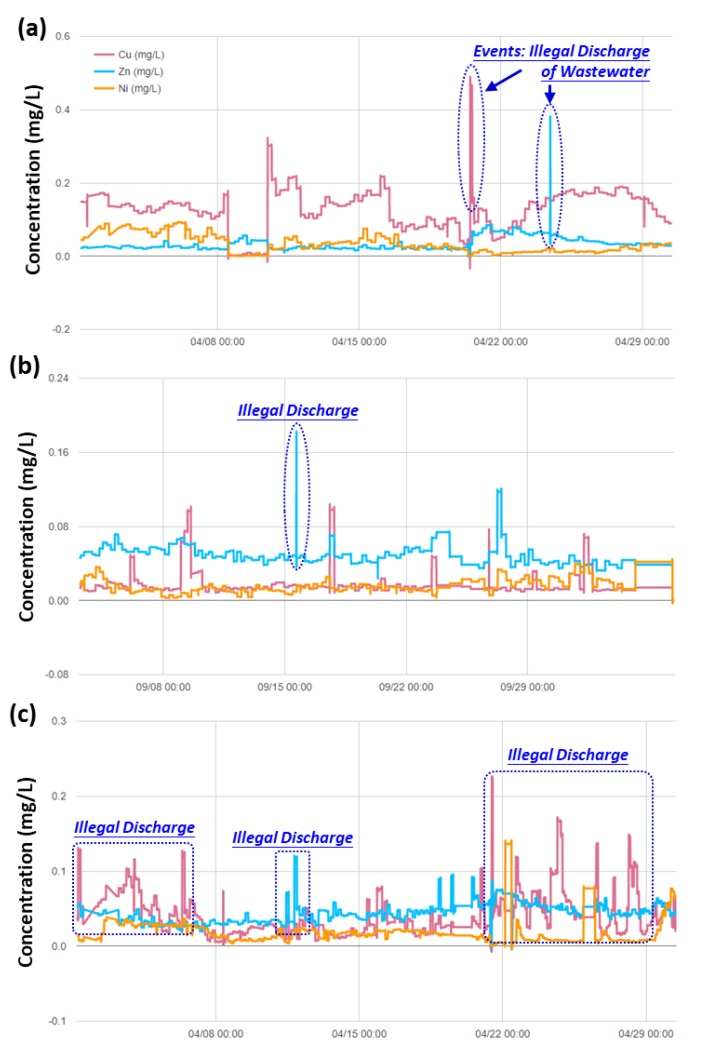
Patterns and trends of heavy metals monitoring (**a**) at the Taoyuan Station A on April 2019, (**b**) at the Taoyuan Station B on September 2018, and (**c**) at the Taoyuan Station C on April 2019.

We have also provided the representative results from the real-time automatic monitoring system of heavy metals, such as Cu, Zn, Ni, and Cr, using stripping voltammetry technique. Figure 7 shows the concentration patterns and trends for different heavy metals (i.e., Cu, Zn, and Ni) at the Taoyuan Station. It was noteworthy to mention that the Taoyuan Station A (Figure 7a) is nearby several large-scale printed circuit board industries. Therefore, the concentrations of copper in water body was found to exceed the discharge standard easily, which could be attributed to the illegal discharge of wastewater. We have also observed several unusual peaks of zinc metals in the waterbody during the monitoring period. For the Taoyuan Station B (Figure 7b), it is located at the Huang Weixi River, the receiving waterbody of the Zhongyu Industrial Park. Despite in close proximity to the industrial park, the average concentration of copper in waterbody still met the water quality standard of irrigation water (copper < 0.2 mg/L [13]). However, we observed several unusual peaks of zinc metals in the waterbody probably because of the illegal discharge of wastewater. The Taoyuan Station C (Figure 7c) is also in close proximity to large-scale printed circuit board industries. According to the real-time automatic monitoring results, we have identified several unusual events where the concentrations of both copper and nickel were higher than that of the background concentration.

**Figure 8:**
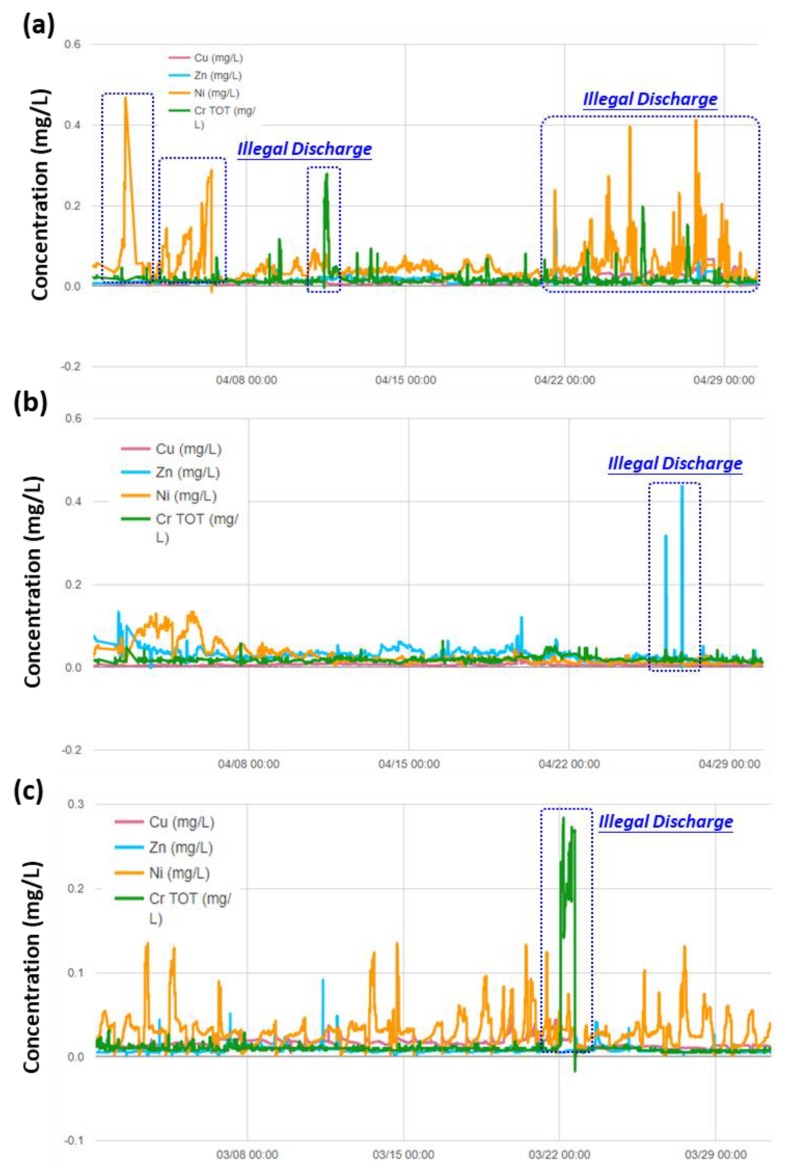
Patterns and trends of heavy metals monitoring (**a**) at Taichung Zhanyiyuan ditch on April 2019, (**b**) at Changhua Xin ditch on April 2019, and (**c**) at Kaohsiung Hankou ditch on April 2019.

Figure 8a–c shows the patterns and trends of representative heavy metals (i.e., Cu, Zn, Ni, and Cr) monitored at Taichung, Changhua, and Kaohsiung, respectively. The results indicated that the water quality at the Taichung monitoring station was significantly influenced by the wastewater discharges from industries, especially for Ni and Cr. Similarly, the water quality at the Changhua monitoring station (Figure 8b) was significantly affected by the upstream metal processing industries, especially for Zn metals. The water quality at the Kaohsiung monitoring station (Figure 8c) also varied remarkably because of the illegal of wastewater discharge from surrounding plants. The concentrations of Ni, Cr, and Zn were found to be unusually high compared to the background concentration.

After continuous operations of the real-time automatic monitoring for years, we summarized our experience and provided several recommendations for operations of the heavy metal monitoring stations, as presented in Table 3. For instance, we noticed that the extent of water quality greatly affected the frequency of maintenances. If the water sample in the field is highly viscous, it increases the frequency of temporary cleaning and polishing of analytical electrodes. Additional pretreatment on water samples might be a solution to avoid the frequent maintenance. Also, the operation and maintenance should be conducted by professional and experienced engineers. Careless operations will increase the frequency and costs of maintenance, and even damage the instrument.

### 3.4. Implications to Ensuring the Safety of Irrigation Water

In this section, we illustrate the implications of the developed automatic real-time monitoring system for ensuring the safety of irrigation water, including (1) integration with automatic sampling for establishing information exchange platform, (2) estimating fluxes of heavy metals to paddy fields, and (3) combining with green technologies for nonpoint source pollution control.

(1) Integration with Automatic Sampling for Establishing Information Exchange Platform.

Table 4 presents the comparison of commonly used stations for on-site water quality monitoring (including heavy metals, pH and EC) and those in the laboratory test. It was noted that the laboratory test with manual sampling of water is usually labor-intensive and time-consuming. In this study, we compiled the commercially available sensors and/or items for measuring water quality to develop an automatic real-time monitoring system, and then installed the developed system in the field for continuous monitoring. In the future, the integration of real-time monitoring and automatic sampling can effectively improve the efficiency of water quality management (in terms of frequency and intensity of sampling and monitoring) and ensure the safety of irrigation water. It was discussed that the system would immediately issue an early warning by notifying the authority manager by short message service and/or email for follow-up decision making processes and actions when a certain water quality parameter exceeds the default threshold. The real-time automatic monitoring system will also allow the remote control and operation of on-site water sampling. Therefore, the costs and time of existing manual sampling systems can be largely reduced.

In addition, the developed water quality monitoring system was combined with the hydrological surveying (obtained from the existing automatic hydrology monitoring) systems to provide an integrated database for establishing the information exchange platform. The information exchange platform compiled the water quality data with hydrological (such as water level and water flow rate) and meteorological (such as rainfall intensity) information. The authority manager can simultaneously access multiple types of information such as hydrology and water quality, and thereby significantly improving the efficiency of decision-making in irrigation management. This information exchange platform also assisted in improving the efficiency of the inspection for the illegal discharge of wastewater in a real time. Moreover, the developed real-time monitoring system and the information exchange platform played a critical role in flood management. The information exchange platform was equipped with the remote-control module, such as the opening and closing gauge, to control the water gate. Thus, it was suggested that the existing system (such as water quality monitoring and water gate management practice) could be integrated with the developed real-time system and information exchange platform to provide an integrated solution for irrigation management in the near future.

(2) Estimating Fluxes of Heavy Metals to Paddy Fields.

The accumulation of heavy metals in paddy soil, especially mercury, cadmium, copper, lead, and chromium, has attracted great attention because of their high toxicity and persistence. The major input sources of heavy metals include irrigation water, fertilizers, and atmospheric deposition [15,16], while the main outputs of heavy metals include runoff water, infiltration, leaching, and plant uptakes [17]. However, only few studies have been carried out for the analysis of fluxes of heavy metals in the soil system. The use of heavy-metal-containing water to irrigate paddy fields will gradually cause a build-up of heavy metals in soil, even if the irrigation water meets the required standard of water quality. With the developed automatic real-time monitoring system, the balance between input and output fluxes of heavy metals (i.e., dynamic mass balance) to paddy soils can be systematically determined via regional field analyses. This can provide useful information regarding the effective accumulation concentration of heavy metals in soils.

(3) Combining with Green Technologies for Nonpoint Source Pollution Control.

The developed automatic real-time monitoring system should be combined with green technologies for nonpoint source pollution control to ensure irrigation water quality. Agricultural nonpoint source pollution, such as cropland runoff, wastes from livestock operations, and aquacultural wastewater, would pose a significant impact to irrigation water and ecosystem quality. The major types of pollutants from agricultural nonpoint source include nutrients (e.g., nitrogen and phosphorus), pesticides (e.g., organophosphate), organics, pharmaceuticals (e.g., antibiotics), pathogens, and even heavy metals. On the flip side, excessive irrigation and extensive usage of fertilizers and pesticides might also result in the nonpoint source pollution [18,19]. These nonpoint sources of pollution can be effectively controlled and remediated by cleaner production and green technologies. For instance, the manure and wastewater from livestock operation can be converted to bioenergy (e.g., biomethane) through various types of green technologies, such as anaerobic digestion [20,21]. With the combination of real-time monitoring system, the physico-chemical properties of the nonpoint source pollution can be efficiently determined to provide useful information for subsequent treatments and/or controls.

## 4. Conclusions

In this study, we have evaluated the performance of the developed automatic real-time monitoring system of water quality for the sake of irrigation management. We have provided several examples (regarding pH and EC) to illustrate the fundamentals and applications of data imputation, and trend and variance analysis at different types of monitoring stations at Taiwan. We have also provided the criteria and principles of determining the thresholds of important water quality parameters based on the past monitoring data. For instance, the threshold of water quality parameters at a ditch-water type station (complex water quality with large variances) could be set as 30‒50% of the 1-h moving average rate of change. The real-time automatic monitoring system also allowed the remote control and operation of on-site water sampling. For the detection of heavy metals, we utilized the stripping voltammetry method to on-site analyze the concentrations of targeted heavy metals in waterbody. The results indicated that the automatic sampling approach was much informative when compared to the manual sampling and was able to reflect the real situation of heavy metals in waterbody. According to the real-time automatic monitoring results, we could also be immediately notified about the unusual events, thereby providing great opportunities to inspect illegal discharge of industrial wastewater. Furthermore, the developed water quality monitoring system was combined with hydrological (e.g., water level and water flow rate) and meteorological (e.g., rainfall intensity) information to serve as the information exchange platform. The authority manager could simultaneously access multiple types of information such as hydrology and water quality, and thereby significantly improved the efficiency of decision-making in irrigation and flood management.

## Figures and Tables

**Table 1:** Specification of commercial sensors used in automatic real-time monitoring system.

Item	Water Quality Parameter			
	pH	Temp.	EC	Heavy Metal
Range of measurement	0‒14	−5 °C~50 °C	0‒100 mS/cm	0.5‒10.0 µg/L
Accuracy	±0.2	±0.1 °C	±1% full scale	0.01 µg/L
Resolution	0.01	0.01 °C	1 mV	−2 V~+2 V (Sensitivity: 1 nA)
Frequency of Data	1 min	1 min	1 min	<30 min
Volume of Feed Water	In-situ	In-situ	In-situ	10 mL
Maintenance	Once a month	Once a month	Once a month	Once a month

**Table 2:** Representative monitoring stations for basic water quality parameters of irrigation water in Taoyuan, Taichung, Changhua, and Kaohsiung. All stations feature automatic sampling function once the water quality exceeds the threshold values.

ID	Irrigation Associations	Station Location	Associated Watershed	Monitoring Items ^a^	Threshold for pH (-)	Threshold for EC (µS/cm)
1	Taoyuan	No. 25-3 River Dam	Sanquaichuo Branch	pH, EC, *T_m_*	6 and 9	750
2		Shuchuo Intake	Shuchuo Drainage	pH, EC, *T_m_*	6 and 9	750
3		Erzhixian	Taoyuan Daquan	pH, EC, *T_m_*, Water Level	6 and 9	750
4		No 6-14 Intake	Laojie Stream	pH, EC, *T_m_*, Water Level	6 and 9	750
5		Qiedong Upstream	Qiedong Stream	pH, EC, *T_m_*, Water Level	6 and 9	750
6		Xiazhongfu Drainage	Xiazhongfu Drainage	pH, EC, *T_m_*, Water Level	6 and 9	750
7		No. 24 Puxin Stream	Puxin Stream	pH, EC, *T_m_*, Water Level	6 and 9	1200
8		No. 25-7 Puxin Stream	Puxin Stream	pH, EC, *T_m_*, Water Level	6 and 9	1200
9		No. 25 River Dam	Sanquaichuo Branch	pH, EC, *T_m_*, Water Level	6 and 9	1500
10		No. 6-5 Intake	Qia Stream	pH, EC, *T_m_*, Water Level	6 and 9	750
11		No. 34 Intake	Xinjie Stream	pH, EC, *T_m_*, Water Level	6 and 9	750
12		Xinjie Upstream	Xinjie Stream	pH, EC, *T_m_*, Cd, Pb, Cu, Ni, Zn	6 and 9	750
13		Xinzhuang No. 3 Bridge	Sanquaichuo Branch	pH, EC, *T_m_*, Cd, Pb, Cu, Ni, Zn	6 and 9	4000
14	Taichung	No. 35 Yuanlizhen Shangan	Xinfugou	pH, EC, *T_m_*	6 and 9	750
15		Datuliaozhenyigei Intake	Dali Stream	pH, EC, *T_m_*, Water Level	6 and 9	750
16		Datuliaozhenyigei Downstream	Dali Stream	pH, EC, *T_m_*, Water Level	6 and 9	750
17		Zhancuoyuanzhen Zhongxing Drainage	Zhongxing Drainage	pH, EC, *T_m_*, Water Level	6 and 9	750
18		Zhancuoyuanzhen	Toubiankeng Stream	pH, EC, *T_m_*, Water Level	6 and 9	750
19		Babaozhen Niuchou Branch	Dajia Stream	pH, EC, *T_m_*, Water Level	6 and 9	750
20		Babaozhen Shangpi Branch	Dajia Stream	pH, EC, *T_m_*, Water Level	6 and 9	750
21		No. 2, 3 Zhancuoyuanzhen	Zhongxing Drainage	pH, EC, *T_m_*, Cd, Pb, Cu, Ni, Zn, Cr	6 and 9	750
22	Changhua	Dongxi Erzhen Jiali Branch	Wu Stream	pH, EC, *T_m_*, Water Level	6 and 9	750
23		Dongxi Erzhen Gongcuo Branch	Wu Stream	pH, EC, *T_m_*, Water Level	6 and 9	750
24		Dongxi Shanzhen Tieshan Branch	Wu Stream	pH, EC, *T_m_*, Water Level	6 and 9	750
25		Si-Liu Guzhen	Wu Stream	pH, EC, *T_m_*, Water Level	6 and 9	750
26		Xinpijiuzhen Intake	Fanyagou	pH, EC, *T_m_*, Water Level	6 and 9	750
27		Xinzhen Intake	Yangzicuo Stream	pH, EC, *T_m_*, Water Level	6 and 9	750
28		Dongxi Shanzhen Midstream	Wu Stream	pH, EC, *T_m_*, Water Level	6 and 9	750
29		Jintunzhen Intake	Shigou Drainage	pH, EC, *T_m_*, Water Level	6 and 9	1500
30		Zhangshui Road	jiu zhuo shui Stream	pH, EC, *T_m_*, Water Level	6 and 9	750
31		Andong Erpai	Andong Erpai	pH, EC, *T_m_*, Water Level	6 and 9	1500
32		Gongcuo Branch	Wu Stream	pH, EC, *T_m_*, Water Level	6 and 9	1200
33		Sanjiachun Drainage	Sanjiachun Drainage	pH, EC, *T_m_*, Water Level	6 and 9	1200
34		Fanyagou Branch Intake	Fanyagou	pH, EC, *T_m_*, Water Level	6 and 9	750
35		Xinzhen Midstream	yang zi cuo Stream	pH, EC, *T_m_*, Cd, Pb, Cu, Ni, Zn, Cr	6 and 9	750
36	Kaohsiung	Hunei Erren Downstream	Erren River	pH, EC, *T_m_* Water Level	6 and 9	1200
37		Weisui Pumping Station	Wujiawei	pH, EC, *T_m_*, Water Level	6 and 9	3500
38		Hankou	District Road	pH, EC, *T_m_*, Cd, Pb, Cu, Ni, Zn, Cr	6 and 9	750

**^a^***T*_m_: Temperature.

**Table 3:** Recommendations for operations of heavy metal monitoring stations.

Item	Guideline	Description
1	Head distance of external autosampler	The head should be 5 m, and the horizontal distance should be 30 m.
2	Avoid use in harsh environments	High humidity and high gas flow rate would easily cause corrosion and failure of the motherboard’s signal collection board.
3	The extent of water quality would affect the maintenance frequency	If the water sample in the field is viscous, it would increase the frequency of temporary cleaning and polishing of analytical electrodes.
4	Sunlight must be sufficient to provide solar energy	If there is a shelter above the solar panel, use the AC power instead.
5	Avoid using in the vibrating environment	Do not place the equipment in a vibrating environment. Vibration would malfunction the equipment’s circuit board of precision.
6	Keep it in a horizontal position, in a customized sun and heat removal device	If the position is not maintained horizontally, the sample will be tilted and the stirring will be uneven, which will cause deviation in the analysis value. It is recommended to use a customized protection box (with double-layer insulation, top ventilation and heat dissipation), being fixed to the horizontal concrete foundation seat. Prevent dumping and theft!
7	Maintenance by professional and experienced engineers	Careless maintenance will increase the frequency and costs of repairs, or even damage the instrument.

**Table 4:** Comparison of commonly used stations for water quality monitoring.

Type	Heavy Metal Monitoring Station (This Study)	Water Quality Environment Monitoring Station	Manual Sampling (Laboratory Test)
Measure	Heavy metals	pH, EC, etc.	Heavy metals, pH, EC, etc.
Power supply	AC 90‒260 V (Solar energy)	AC 110/240 V	-
Types of water body sample	Rivers, lakes, groundwater	Rivers, lakes, groundwater	Rivers, lakes, groundwater
Accuracy of measure	High	High	Highest
Price of equipment (US $)	7900	11,700	Depending on the test item
Labor costs	Low	Low	High
Maintenance	Once a month	Once a month	-

## Supplementary Materials

The following are available online at https://www.mdpi.com/1660-4601/17/3/737/s1, Figure S1. Typical voltammetric spectrum for monitoring heavy metals in water. The measured current can be used to determine the concentration of metals, and the potential corresponds to the types of metals in water. Figure S2. Representative photos of automatic monitoring stations at (a) Taoyuan, (b) Changhua, and (c) Kaohsiung. Figure S3. Improvement of the portable automatic monitoring device for basic water quality parameters.
