# Supplementary material for: Establishment of an Automatic Real-Time Monitoring System for Irrigation Water Quality Management
Source: Int J Environ Res Public Health. 2020 Jan 23;17(3):737. doi: 10.3390/ijerph17030737 (PMC7038173; doi:10.3390/ijerph17030737)
Supplement: Supplementary file 1 [file ijerph-17-00737-s001.pdf]

Supporting Information

Establishment of an Automated Real-Time Monitoring System for Irrigation Water Quality Management

Wei-Jhan Syu, Tsun-Kuo Chang, Shu-Yuan Pan*

Department of Bioenvironmental Systems Engineering, National Taiwan University, Taipei City, 10617 Taiwan, ROC

* Corresponding author. *E-mail address*: sypan@ntu.edu.tw (S.Y.P.).

Numbers of Pages: 4

Numbers of Figures: 3

Numbers of Tables: 0

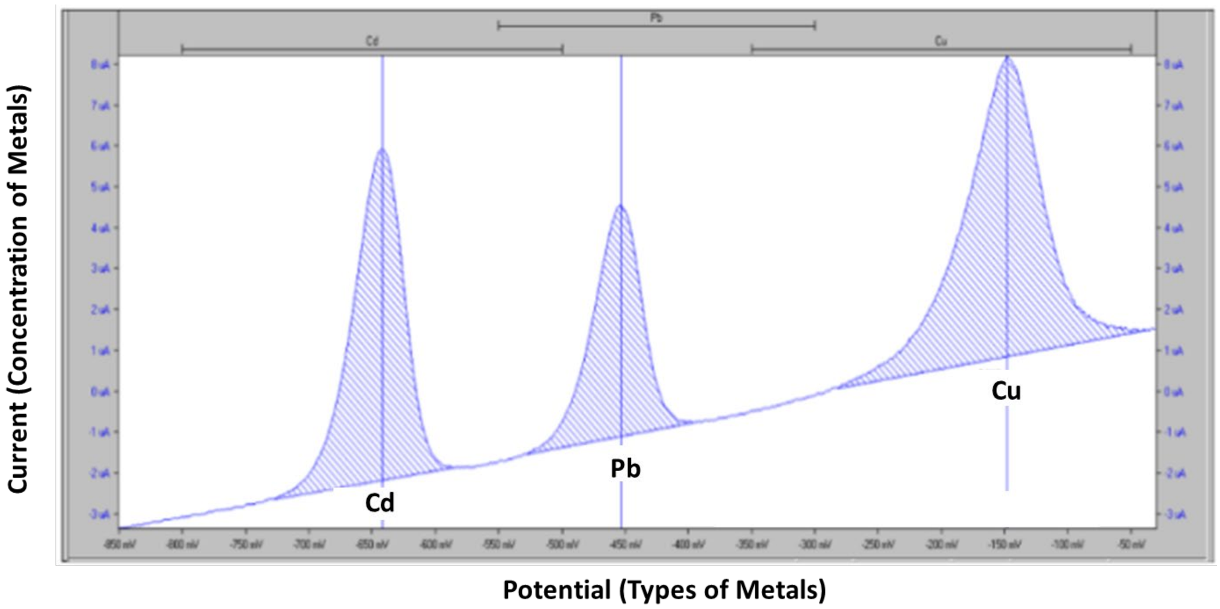

Figure S1. Typical voltammetric spectrum for monitoring heavy metals in water. The measured current can be used to determine the concentration of metals, and the potential corresponds to the types of metals in water

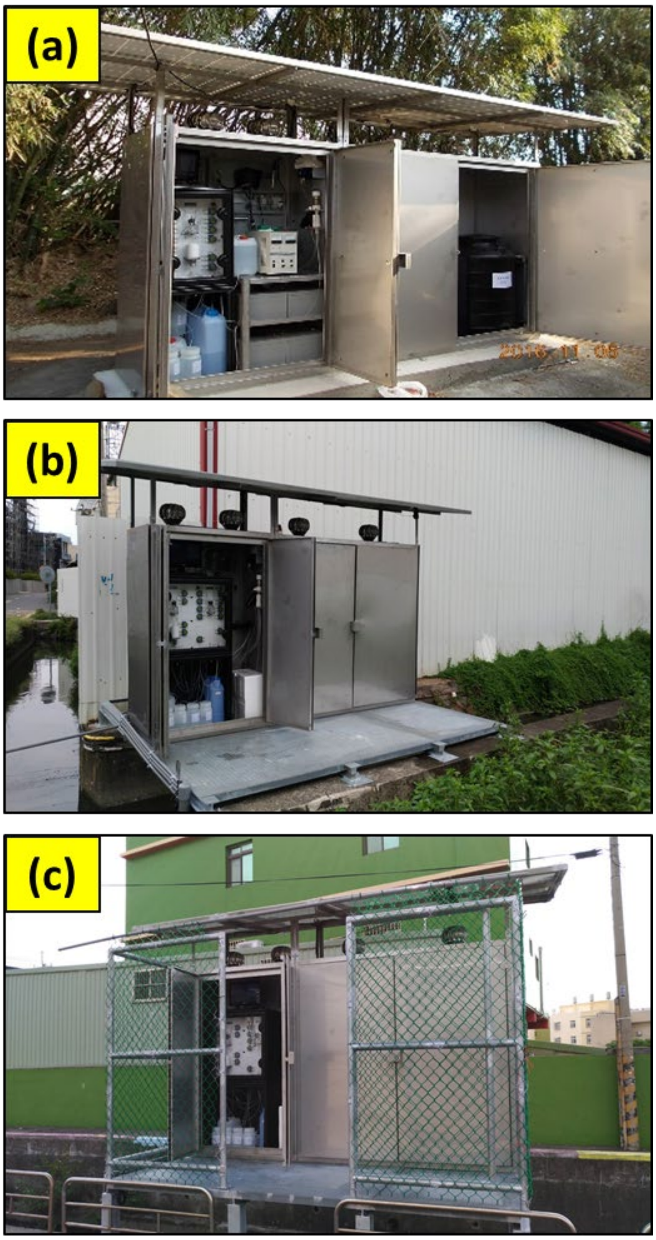

Figure S2. Representative photos of automatic monitoring stations at (a) Taoyuan, (b) Changhua, and (c) Kaohsiung.

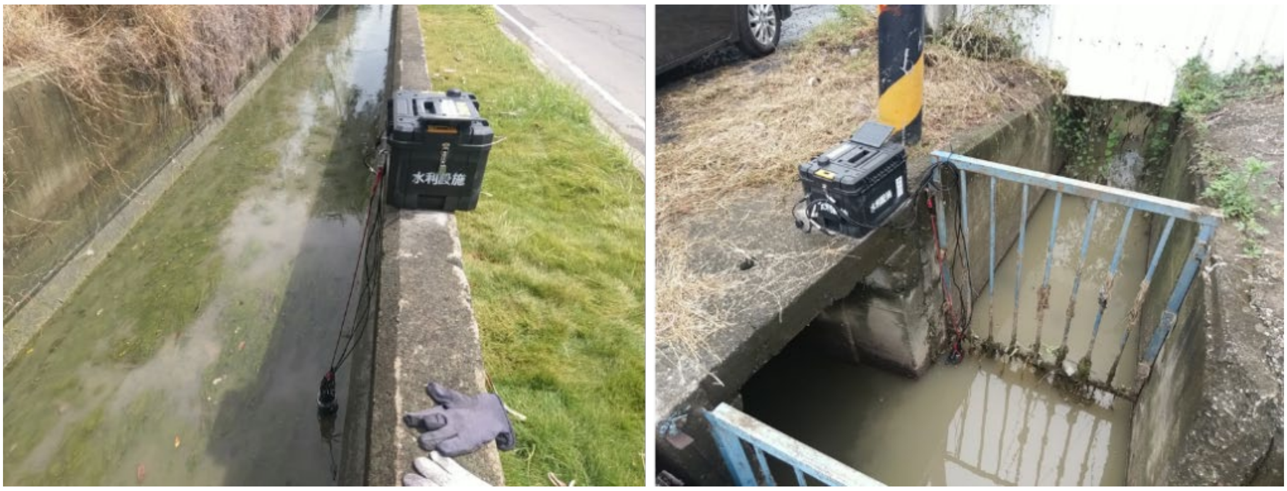

Figure S3. Improvement of the portable automatic monitoring device for basic water quality parameters.
